# Assessment on Agricultural Drought Vulnerability and Spatial Heterogeneity Study in China

**DOI:** 10.3390/ijerph18094449

**Published:** 2021-04-22

**Authors:** Hongpeng Guo, Jia Chen, Chulin Pan

**Affiliations:** College of Biological and Agricultural Engineering, Jilin University, 5988 Renmin Street, Changchun 130012, China; ghp@jlu.edu.cn (H.G.); chenjia19@mails.jlu.edu.cn (J.C.)

**Keywords:** agricultural drought vulnerability, spatial heterogeneity, entropy weight method, contribution model, China

## Abstract

Reducing drought vulnerability is a basis to achieve sustainable development in agriculture. The study focuses on agricultural drought vulnerability in China by selecting 12 indicators from two aspects: drought sensitivity and resilience to drought. In this study, the degree of agricultural drought vulnerability in China has been evaluated by entropy weight method and weighted comprehensive scoring method. The influencing factors have also been analyzed by a contribution model. The results show that: (1) From 1978 to 2018, agricultural drought vulnerability showed a decreasing trend in China with more less vulnerable to mildly vulnerable cities, and less highly vulnerable cities. At the same time, there is a trend where highly vulnerable cities have been converted to mildly vulnerable cities, whereas mildly vulnerable cities have been converted to less vulnerable cities. (2) This paper analyzes the influencing factors of agricultural drought vulnerability by dividing China into six geographic regions. It reveals that the contribution rate of resilience index is over 50% in the central, southern, and eastern parts of China, where agricultural drought vulnerability is relatively low. However, the contribution rate of sensitivity is 75% in the Southwest and Northwest region, where the agricultural drought vulnerability is relatively high. Among influencing factors, the multiple-crop index, the proportion of the rural population and the forest coverage rate have higher contribution rate. This study carries reference significance for understanding the vulnerability of agricultural drought in China and it provides measures for drought prevention and mitigation.

## 1. Introduction

Drought occurs frequently in China and there has been a long history of these occurrences. From 206 BC to 1949, 1056 droughts occurred in China [1]. From 1971 to 2016, the average annual disaster rates of droughts in Heilongjiang, Jilin, Liaoning, and Inner Mongolia Autonomous Region were 19.4%, 23.6%, 25.4% and 29.8%, respectively. The average annual disaster rates of droughts in Anhui, Hebei, Henan, Jiangsu, and Shandong provinces were 11.5%, 20.4%, 16.2%, 8.9%, and 18.3%, respectively [2]. The Ministry of Emergency Management of the People’s Republic of China has notified that from July to November of 2019, droughts had affected a total of 1174 thousand hectares of crops in Jiangxi and Anhui provinces, resulting in a direct economic loss of 8.8 billion yuan [3]. From January to April of 2020, 2.433 million people had been affected in 81 counties of 16 cities (prefectures) in Yunnan Province. A total of 662 thousand people had requested for life assistance due to droughts, and 534 thousand hectares of crops were affected, leading to direct economic loss of 1.41 billion yuan [4].

Drought is considered as a slow-moving natural disaster that causes severe damage to water resources and to agriculture [5]. The characteristics of drought include, but are not limited to, high frequency, long duration, and large area being influenced [6]. Agricultural drought is a crucial part of drought and it refers to the situation where agricultural production is sensitive and vulnerable to drought stress [7]. Agriculture utilizes natural resources directly and it is also a national anchoring industry. Agriculture is less capable of resisting and dealing with disasters. The resistance and handling capacity of agriculture to disasters is low so the adverse impact on agricultural production is most severe when drought occurs. In the same way, droughts can be intensified by poor land management [8]. Therefore, the situation of agriculture and the extent of drought affect each other. According to the Assessment Report of the AR5 Climate Change 2014: Impacts, Adaptation, and Vulnerability: Vulnerability encompasses a variety of concepts and elements including sensitivity or susceptibility to harm and lack of capacity to cope and adapt [9]. Taking the initiative via human activity is an effective way to alleviate the loss caused by a drought disaster [5]. So, measuring agricultural drought vulnerability is a prerequisite for targeting interventions to improve and sustain the agricultural performance of both irrigated and rain-fed agriculture [10].

Climate change has an increasing impact on production and people’s lives. In recent years, the topic of vulnerability to agricultural drought has gradually become the focus and research hotspot of scholars around the world.

Yi (2010) evaluated the agricultural droughts in Dalian, China. Ten evaluation indexes such as irrigation index, population density and proportion of paddy areas were selected [11]. Yuan (2016) proposed a comprehensive index of regional drought vulnerability that includes exposure, sensitivity, and adaptability [12]. The establishment of evaluation indicators cannot be applied to all since it is highly subjective to regional characteristics. However, different indexing systems provide more research possibilities in the field of drought vulnerability.

Yan (2012), Pang et al. (2013), Farhangfar et al. (2015), Liu et al. (2015), and others conducted quantitative evaluation on drought vulnerability of maize and wheat and obtained the severity and spatial changes of crops at different growth stages [13,14,15,16]. Kim et al. (2018) used multivariate statistical analysis method to assess the agricultural vulnerability to droughts in South Korea and the results showed that the Chungchongnam-Do area was most vulnerable [17]. Lestari et al. (2018) used Arc GIS spatial overlay analysis to evaluate the agricultural drought vulnerability of Semarang Port City in India. The results showed that high vulnerability in six villages, medium vulnerability in seven villages, and low vulnerability in three villages [18]. Based on super sufficiency DEA, Huang et al. (2019) evaluated the agricultural drought vulnerability of Hetao Irrigation Area in Inner Mongolia and the results showed that the drought vulnerability in the eastern part of Hetao Irrigation Area was much higher than that in the western part [19]. Frischen et al. (2019) combined the result from spatial analysis of expert consultation and determined the drought vulnerability of Zimbabwe’s agricultural system. The results showed that the country’s drought vulnerability and the degree of impact vary greatly. The northern and southern part of Matabeleland, a province in southwestern part, have higher vulnerability level [20]. Das et al. (2019) used Savitzky and Golay filtering methods to study the agricultural drought situation and vulnerability in India from 1982 to 2015. Results showed that the vulnerability of drought will continue to decrease over time [21]. On the basis of selecting the research areas and constructing the evaluation index system, scholars have adopted different methods to evaluate the agricultural vulnerability to droughts. For example: Data envelopment analysis [22,23], analytic hierarchy process [24,25,26,27,28], principal component analysis [29,30], entropy weight method [31,32,33], etc. STATA [34,35], ArcGIS [36,37,38] and other software have also been used to construct an evaluation model for quantitative analysis.

Rojas et al. (2011) and Zhang et al. (2016) used remote sensing technology to monitor and predict agricultural drought [39,40]. Guo et al. (2016) proposed a new method (vulnerability surfaces) for assessing vulnerability quantitatively and continuously by including the environmental variable as an additional perspective on exposure and assessed global drought risk of maize based on these surfaces [41]. Chen et al. (2017) and Zeng et al. (2019) conducted drought risk assessment on Yunnan Province and Gansu Province respectively [42,43]. All the above studies have provided scientific methods for drought risk assessment and they have since enriched the assessment system for agricultural drought vulnerability.

Basing on a wide range of research areas and research methods, there exists the differences in the natural geographical environment, economic and social conditions, which has led to different influencing factors and various degrees of agricultural drought vulnerability. For example: Zarafshani et al. (2012) argued that the vulnerability of wheat farmers in the western part of Iran is mainly affected by economical, socio-cultural, psychological, technological, and infrastructural factors [44]. Wu et al. (2017) believed that the water shortage rate and irrigation level in the growing season were the main factors affecting the vulnerability level of regional agricultural drought [45]. Kamali et al. (2019) believed that the fertilization level is an important factor affecting the vulnerability of crop to drought in sub-Saharan Africa. Generally, countries with a higher food production index and better infrastructure perform better in terms of withstanding drought [46].

To sum up, there are two methods namely qualitative research and quantitative research on agricultural drought vulnerability. Existing research on agricultural drought vulnerability in China mainly focused on certain regions for quantitative research [7,14,32,37,45,47,48,49,50,51]. There were only a few studies on the overall assessment of agricultural drought vulnerability and among those the research objects, conclusions and countermeasures are limited. 

Therefore, this paper focuses on the agricultural drought vulnerability in China. Based on literature review and relative theories, the paper first constructs the vulnerability evaluation index system of agricultural drought. Then the paper uses entropy weight method, weighted comprehensive scoring method as well as k-means clustering algorithm to evaluate and categorize the vulnerability of agricultural drought in China. Finally, using the contribution model to analyze the influencing factors and the degrees of agricultural drought vulnerability in China, this paper proposes countermeasures to reduce agricultural drought vulnerability in China. In one aspect, the paper carries theoretical value for enriching vulnerability research. It is also conducive to a better understanding of drought conditions and influencing factors in various regions of China. In another aspect, the empirical analysis provides the basis for the government to formulate corresponding policies, to reduce losses caused by disasters, and to promote the sustainable development of agriculture in China.

## 2. Materials and Methods

### 2.1. Research Area Overview

The People’s Republic of China is located in East Asia and to the west coast of the Pacific Ocean. Liberated on 1 October 1949, China’s capital city is Beijing and the provincial administrative divisions are divided into twenty-three provinces, five autonomous regions, four municipalities, and two special administrative regions. China’s land area is about 9.6 million square kilometers. China is the world’s second largest economy, the world’s largest industrial country, and the world’s largest agricultural country. At the end of 2019, the total population of mainland China was more than 1.4 billion.

The terrain is high in the West and low in the East. Mountains, plateaus, and hills account for estimated 67% of the land area, basins, and plains account for around 33% of the total land area. The climate condition is complex and diverse.

Looking at the situation and distribution of China’s agricultural natural resources as a whole, the light and heat conditions are superior. However, there is a great regional differences of dry and wet conditions. The total amount of river runoff is large; however, the coordination and distribution of soil and water is not even. The absolute amount of land resources is large; however, the land occupied per capita is small. Agriculture still serves as the basic industry of China’s national economy.

### 2.2. Establishment of Indicator System and Data Sources

The establishment of evaluation index system is the prerequisite for evaluating agricultural drought vulnerability. Vulnerability is the root cause of drought disasters, which results from the interaction of natural environment and social economy system as well as the interactions of sensitivity and resilience in a certain space. Therefore, following the principles of science, comprehensiveness, pertinence, quantification, and availability of data [47], we select two first-level indicators, namely, sensitivity and resilience and 12 second-level indicators to conduct an evaluation on 31 provincial administrative units (except for Hong Kong, Macao, and Taiwan) in China to establish an indicator system (as shown in Table 1). The larger the indicator, the larger the vulnerability of agricultural drought. Hence, it is a positive indicator. On the contrary, it would be a negative indicator.

Sensitivity is the sum of all kinds of natural and social factors that would cause or aggravate drought and its impact on agricultural drought vulnerability is negative. That means the higher the sensitivity, the greater the vulnerability of agricultural drought. It includes agriculture in GDP proportion, multiple-crop index, rural population proportion, annual average temperature, annual sunshine duration, and annual precipitation.

Higher proportion of agriculture in GDP means that farmers rely heavily on agricultural income which is highly dependent on natural conditions. So the vulnerability of agricultural drought will increase. The higher the multiple-crop index, the more water the crop would need to grow. As a result, drought vulnerability will increase. The most severely impacted population at the time of drought is the agricultural population. Therefore, when the proportion of rural population increases, the degree of vulnerability will also increase. Moreover, higher the temperature and longer sunshine hours will lead to the increase of evaporation, and hence the agricultural drought vulnerability will increase together. Precipitation is the main factor affecting the growth of crops. The precipitation index can reflect the meteorological conditions of crops in this region and the impact of precipitation on vulnerability is negative.

Resilience refers to the ability of human society to prepare for, to respond to, and to recover from, disasters. It has a positive impact on agricultural drought vulnerability. That means the stronger the resilience, the lower the drought vulnerability. It includes forest coverage rate, net income per capita of rural residents, food production per capita, real GDP per capita, effective irrigation rate, and agricultural fertilizer per unit area.

The forest coverage rate reflects a country’s (or region) actual level of forest resources and forestry possession. Net income per capita of rural residents reflects the group of people’s economical ability to withstand and to resist drought. The higher the net income per capita of rural residents, the weaker the threats of agricultural drought. Food production per capita reflects the level of agricultural productivity. Real GDP per capita reflects the level of social and economic development. When the index is bigger, it means that the social and economic development level and the ability to withstand disasters is high. The effective irrigation rate reflects the degree of water conservancy and irrigation capacity. The increase of the amount of agricultural fertilizer per unit area is beneficial to enhance soil fertility, to improve soil structure and to increase the efficiency of land usage. The above indicators constitute the resilience of the agricultural system.

The agriculture in GDP proportion, the rural population proportion, the net income per capita of rural residents, the food production per capita, and the real GDP per capita affect the agricultural drought vulnerability from the economic and social perspectives. The multiple-crop index, the effective irrigation rate and the agricultural fertilizer per unit area affect the vulnerability of agricultural drought from the perspective of agricultural technology. The forest coverage rate, annual average temperature, annual sunshine duration, and precipitation affect the vulnerability of agriculture to drought from the perspective of natural conditions.

The indicator data in this paper comes from the website of the National Bureau of Statistics [52] and the China Meteorological Administration [53]. The annual precipitation, annual sunshine duration and annual average temperature are obtained from annual observations from 613 weather stations nationwide from China Meteorological Administration data network. In addition to the forest coverage rate, net income per capita of rural residents and real GDP (Gross Domestic Product) per capita can be directly obtained, other indicators need to be calculated. The descriptive statistical results of the complete sample are shown in Table 2.

**Table 1:** Index system and source of China’s agricultural drought vulnerability assessment.

Indicators and Units	Calculation Formula	Source
Agriculture in GDP proportion (%)	Agricultural output value/GDP	[51,54]
Multiple-crop index (%)	Cultivated area of crops/Total cultivated area	[49]
Rural population proportion (%)	Rural population/Total population	[51,54]
Annual average temperature (°C)	Annual average value of each meteorological station	[32]
Annual sunshine duration (h)	Annual average value of each meteorological station	[55]
Annual precipitation (mm)	Annual average value of each meteorological station	[51,54,56]
The forest coverage rate (%)	Available directly	[56,57]
Net income per capita of rural residents (yuan/per)	Available directly	[22,58,59]
Food production per capita (kg/per)	Food production/Total population	[49]
Real GDP per capita (yuan/per)	Available directly	[32,51,59]
The effective irrigation rate (%)	Effective irrigation area/Total cultivated area	[31,56]
Agricultural fertilizer per unit area (ton/hm^2^)	Amount of fertilizer used/Total cultivated area	[32]

### 2.3. Data Processing

From Table 1, each indicator has different dimensions; hence, direct comparison is not possible. Therefore, it is necessary to carry out the dimensionless standardization of each indicator. The positive and negative indicators have different influence directions on agricultural drought vulnerability so the treatment methods should be different.

Suppose there are k provinces, n years and m evaluation indicators; then $X_{\theta ij}$ represents the j indicator value of province i in year θ. The normalized value after treatment is expressed as $S_{\theta ij}$ (0 < $S_{\theta ij}$ < 1). $X_{\min}$ is the minimum value of the *j* indicator and $X_{\max}$ is the maximum value of the *j* indicator.

Positive indicator:

(1) $$S_{\theta ij}=\frac{X_{\theta ij}-X_{min}}{X_{max}-X_{min}}$$

Negative indicator:

(2) $$S_{\theta ij}=\frac{X_{max}-X_{\theta ij}}{X_{max}-X_{min}}$$

### 2.4. Improved Entropy Weight Method

There are two methods to determine the weight: subjective weight method and objective weight method. This paper chooses the entropy weighting method (one of the objective weighting methods) for indicator weighting, which overcomes the subjective arbitrariness of the subjective weighting method and makes the weighting more scientific. The improved entropy weighting method has the following methods and steps [60,61]:

Build the matrix $Y_{\theta ij}$:

(3) $$Y_{\theta ij}=\frac{S_{\theta ij}}{\sum_{\theta }\sum_{i}S_{\theta ij}}$$

Calculate indicator information entropy $e_{j}$:

(4) $$e_{j}=-\frac{1}{\ln kn}\sum_{\theta }\sum_{i}Y_{ij}\ln(Y_{\theta ij})$$

Find indicator difference coefficient (redundancy) $g_{j}$:

(5) $$g_{j}=1-e_{j}$$

The weight of each indicator $w_{j}$:

(6) $$W_{j}=\frac{g_{j}}{\sum_{j}g_{j}}$$

### 2.5. Vulnerability Assessment Model

This paper chooses the weighted comprehensive scoring method and uses $V_{\theta i}$ to represent the degree of vulnerability. The improved vulnerability assessment model of agricultural drought in China is as follows:

(7) $$V_{\theta i}=\sum_{j}\left(W_{j}\times S_{\theta ij}\right)$$

### 2.6. K-Means Clustering Algorithm

According to the above steps, to calculate the degree of vulnerability of the target year of China’s agricultural drought in various regions and put them in ascending order. After that, to use k-means clustering algorithm in Stata to grade the vulnerability of China’s agricultural drought disaster [48,62].

Algorithms usually use Euclidean distance to calculate the distance between samples. The calculation formula is as follows:

(8) $$d(x,y)=\sqrt{\sum_{i=1}^{n}{\left(x_{i}-y_{i}\right)}^{2}}$$

Suppose the class center of the k category is $center_{k}$, then the formula of $center_{k}$ is updated as follows:

(9) $$center_{k}=\frac{1}{\left|c_{k}\right|}\sum_{x_{i}\in c_{k}}x_{i}$$

The clustering algorithm requires continuous iteration to re-classify and update $center_{k}$ value. Whenever the maximum number of iterations has been reached or the objective function is less than the threshold value, the iteration ends. The objective function is as follows:

(10) $$J=\sum_{k=1}^{k}\sum_{x_{i}\in c_{k}}d(x_{i},center_{k})$$

### 2.7. Contribution Model

The main contributing factors of agricultural drought vulnerability in China are analyzed by contribution model. $R_{j}$ is the weight of the j criterion level indicator; $C_{ij}$ is the contribution degree of the j indicator factor to the vulnerability of the i evaluation object; $U_{r}$ represents the contribution of the first level indicators to vulnerability; $F_{j}$ is the weight of single indicator to total target; $I_{ij}$ is the indicator membership degree (that is to say the proportion of Single factor indicator accounts for in vulnerability results. In the obstacle degree model, the indicator deviation degree is the difference between the individual index factor evaluation value and 100%. Therefore, the factor membership in the contribution degree model is the single indicator factor evaluation value ratio 100%) [32].

(11) $$F_{j}=R_{j}\times W_{j}$$

(12) $$I_{ij}=1-S_{\theta ij}$$

(13) $$C_{ij}=\frac{F_{j}\times I_{ij}}{\sum_{j}(F_{j}\times I_{ij})}$$

(14) $$U_{r}=\sum C_{ij}$$

## 3. Results and Discussion

### 3.1. Agricultural Drought Vulnerability in China

According to Formulas (1) and (2), after the data is being nondimensionalized and standardized, we use the calculation steps of the entropy weight method (Formulas (3)–(6)) to calculate the weight of each indicator, which is shown in Table 3.

It can be seen that, for the two first-class indicators, sensitivity index weight is 0.594 and resilience index weight is 0.406. Among them, multiple-crop index, annual average temperature, the forest coverage rate, the effective irrigation rate, and agriculture in GDP proportion have higher weight of over 0.1. Since the weight of agricultural in GDP proportion is 0.099, which is very close to 0.1, we also put significant important over this index.

According to Formula (7), the agricultural drought vulnerability degree of each region in 1978, 1983, 1988, 1993, 1998, 2003, 2008, 2013, and 2018 have been calculated and shown in Table 4.

It can be noticed that from 1978 to 2018, the vulnerability of agricultural drought in China has decreased year by year. Agricultural drought vulnerability in Gansu, Ningxia, Guizhou, and Tibet is relatively high with an average value of more than 0.648. Agricultural drought vulnerability in Shanghai, Beijing, and Zhejiang is low with the average value less than 0.47.

#### 3.1.1. Classification of Agricultural Drought Vulnerability

In order to accurately classify China’s agricultural drought vulnerability, according to Formulas (8)–(10) by using k-means clustering algorithm in Stata, the China Agricultural Drought Vulnerability Index (ADVI) is divided into four ranges between 0 to 1 and they are shown in Table 5.

#### 3.1.2. Spatial Distribution and Evolution

**Figure 1:**
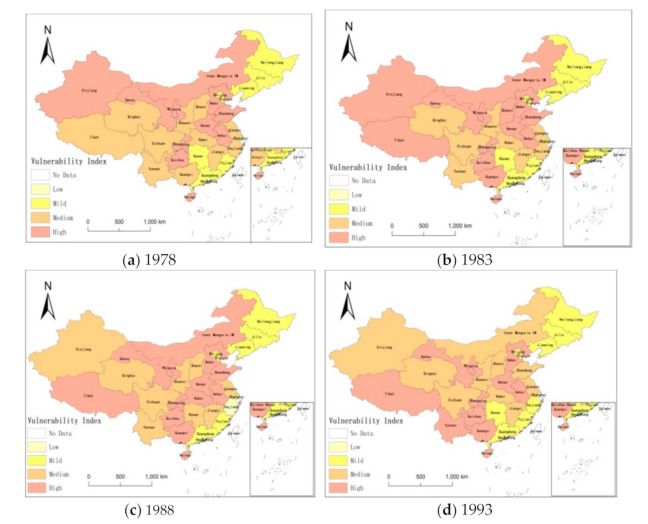

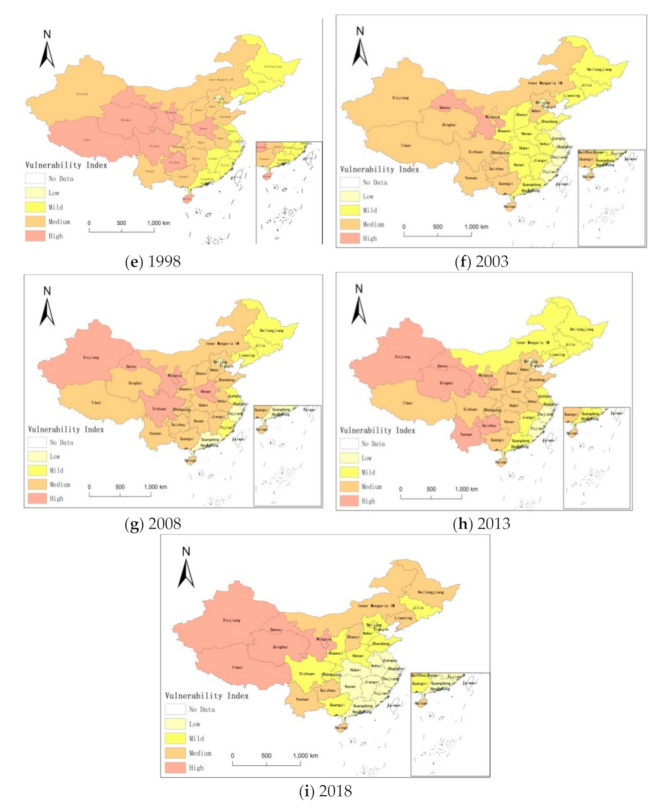
Spatiotemporal evolution of agricultural drought vulnerability in China (**a**–**i**).

According to the classification of agricultural drought vulnerability in Table 5, in order to express the results more clearly, this study uses ArcGIS to display the research results. The assessment results of agricultural drought vulnerability in China are shown in Figure 1a–i.

It can be seen that the vulnerability of agricultural drought in China has changed significantly over time: from 1978 to 2018, the number of provinces and cities in low and mild vulnerability state has been increasing.

From 1978 to 2018, the spatial distribution pattern of agricultural drought vulnerability in China was obvious:

(1) The overall agricultural drought vulnerability in China is 0.569, which is at a moderate fragile level. This is in line with the characteristics of frequent droughts and serious losses in China [63,64,65].
(2) Highly vulnerability level: (0.628 < ADVI < 1) Over time, the number of cities in highly vulnerable areas has decreased, which mainly included Xizang, Guizhou, Ningxia, Gansu, etc. Among them, Gansu, Ningxia have higher vulnerability to drought, which is consistent with the research results of other scholars [59,66]. Firstly, most of these areas have complex terrain conditions and less precipitation. Drought is their main natural feature. Secondly, the region is less developed compared with other regions and real GDP per capita is low while agriculture in GDP proportion and rural population proportion is high. It means that farmers are highly dependent on agricultural and natural conditions. With high sensitivity and weak resilience when drought occurs, the number of highly vulnerable provinces and cities are inevitably high.
(3) Middle vulnerability level: (0.552 < ADVI < 0.628) The number of provinces and cities in this region is stable and it accounts for nearly half of the total number of provinces and cities in China and most of them are concentrated in Central China. It included Inner Mongolia, Sichuan, Hebei, Anhui, etc. Most of them are important grain production bases in China and major agricultural provinces. Agriculture in GDP proportion, multiple-crop index and rural population proportion are high. It reflects that the region has a strong dependence on agriculture with high land utilization rate and heavy water demand.
(4) Low and mild vulnerability level: (0 < ADVI < 0.552) Although there has seen a small fluctuation in the number of slightly vulnerable provinces and cities, the overall trend shows a stable and marginal increase. This is consistent with the research results of some scholars [22,67]. The provinces and cities in this region such as Shanghai, Zhejiang, Beijing, and Tianjin have a high level of economic development. Their high real GDP per capita gives them better response ability and post disaster recovery ability when disasters occur. At the same time, those provinces and cities tend to have a small agricultural planting area multiple-crop index, agriculture in GDP proportion and rural population proportion are also low. When we turn to those provinces and cities in the Northeast China like Heilongjiang, Jilin, and Liaoning, their land is sparsely populated and the food production per capita is high. They also have high latitude, low average temperature, and less evaporation. The annual sunshine duration is long and the crops normally harvest once a year. With lower multiple-crop index, the water demand is lesser and the sensitivity of disaster is weak.

### 3.2. Analysis on the Influencing Factors of Agricultural Drought Vulnerability in China

#### 3.2.1. Factor Contribution Analysis of First Level Index

**Figure 2:**
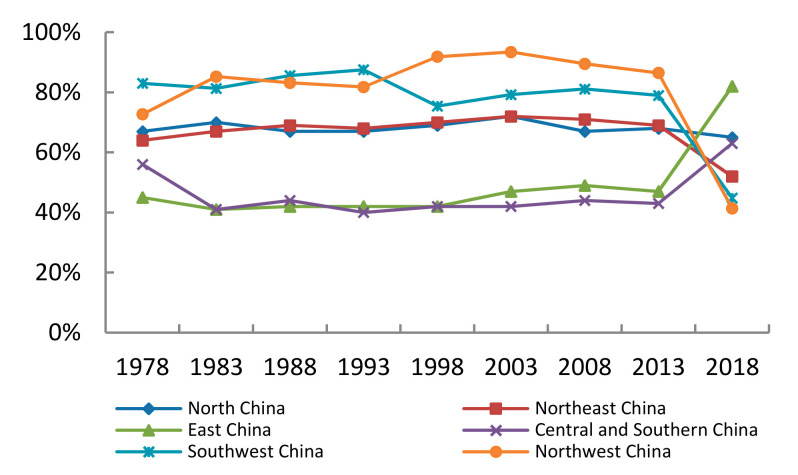
Changes in the contribution of sensitivity indicators.

**Figure 3:**
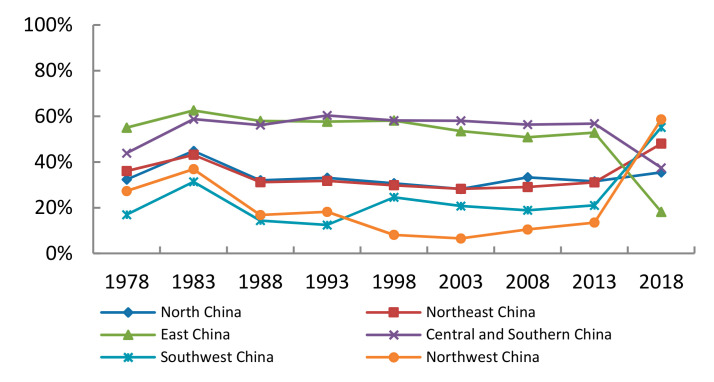
Changes in the contribution of resilience indicators.

According to the research results of agricultural drought vulnerability assessment in China, it can be noticed that the distribution of provinces and cities in different vulnerability levels has certain regional characteristics. Therefore, this paper studies the influencing factors of vulnerability in different regions. It adopts China’s six geographic regions: North China, Northeast China, Northwest China, East China, Central and Southern China, and Southwest China. According to the Formulas (11)–(14), the contribution of sensitivity and resilience is shown in Figure 2 and Figure 3.

As shown in Figure 2, looking at the trends as a whole, during the period of 1978–2013, the contribution of sensitivity indexes in different regions was relatively stable. From 2013 to 2018, except for North China, the contribution ratio of sensitivity indicators in other regions changed dramatically.

The contribution of sensitivity indicators in Northeast China, Northwest China, and Southwest China have declined. Possible reasons are as follows: agriculture in GDP proportion, multiple-crop index, and annual sunshine duration has decreased drastically while annual precipitation has increased significantly. Farmers in these regions have become less dependent on agricultural income. Lower land use has reduced water demand, and hence there is less evaporation.

The contribution of sensitivity indexes in East China and Central South have increased significantly. Possible reasons are as follows: agriculture in GDP proportion and multiple-crop index have increased while land use in the region has improved and water demand has increased.

As shown in Figure 3, looking at the trends as a whole, during the period of 1978–2013, the contribution of resilience indexes in different regions was relatively stable. From 2013 to 2018, except for North China, the contribution ratio of resilience indicators in other regions changed dramatically.

The contribution of resilience indicators in Northeast China, Northwest China, and Southwest China have increased. I think the possible reasons are as follows: the contribution of forest coverage rate, food production per capita, and agricultural fertilizer per unit area have increased while net income per capita of rural residents has declined. The region is less dependent on agriculture, needs to improve its ability to conserve water, and is less able to cope with disasters and recover from disasters.

The contribution of resilience indicators in East China, Central China, and South China have decreased evidently. Possible reasons are as follows: the forest coverage rate, the effective irrigation rate and agricultural fertilizer per unit area have increased. The region’s ability to conserve water is better, so the recovery capacity after the disaster is improved.

On the whole, the contribution of resilience in East China, Central, and Southern China is more than 50%, which is greater than the contribution of sensitivity. Cities in the central and southern region: the Yellow River valley passes through Henan, and the Yangtze River valley passes through Hubei and Hunan. Guangxi, Guangdong, and Hainan are adjacent to the sea. Therefore, the relative abundance of water resources and the contribution of sensitivity indicators are less. East China includes Shanghai, Jiangsu, Zhejiang, Anhui, Fujian, Jiangxi, and Shandong. These several provinces and cities are adjacent to the sea, or within the territory of the river flow through, and the level of economic development is at the forefront of China. Therefore, the contribution degree of resilience index is relatively high.

Northwest China, Northeast China, North China, and Southwest China have higher sensitivity contributions than that of resilience. The sensitivity contribution of the Southwest China and Northwest China is as high as 75%. Desert is widespread in the northwest and annual precipitation about 200–400 mm. Deep inland and blocking the arrival of moist air. Northeast China is an important grain production base with a large area of cultivated land. Compared with the coastal areas, its economic development level is not high.

#### 3.2.2. Factor Contribution Analysis of Secondary Index

Select the top four indicators of contribution among the 12 indicators and the calculated results are shown in Table 6.

On the whole, referring to the calculation results above, it can be noticed that the contribution factors of agricultural drought vulnerability in China mainly focus on sensitivity. Among them, A2 (multiple-crop index) and A3 (rural population proportion) are more important. It shows that these two indicators have a greater impact on the vulnerability of agricultural drought. We should sustainably reduce the land utilization rate, reduce the water demand, strengthen the vocational skills training of rural residents, supervise and protect the legitimate rights and interests of migrant workers, and promote the transfer of rural population to cities. Hence, the proportion of rural population can be effectively reduced, and the vulnerability of agricultural drought can also be mitigated.

Among the indexes of resilience, B1 (the forest coverage rate) and B4 (real GDP per capita) are more important. According to the data from the Ninth National Forest Resources Inventory, China’s forest coverage rate is still lower than the world average level. Strengthening afforestation is highly effective for soil and water conservation, hence reducing water evaporation and improving the forest coverage rate. The adverse impact from drought can also be reduced significantly. Similarly, the higher the real GDP per capita, the easier the recovery would be after droughts. The GDP per capita China is still relatively low in the worldwide spectrum, although China’s total domestic GDP ranks No. 1 in the world.

The factor with the least contribution is B3 (Food production per capita). China has a large planting area of crops with high and stable grain yield, so it has little impact on agricultural drought vulnerability.

## 4. Conclusions, Limitations, and Future Research

The paper uses entropy weight method, weighted comprehensive scoring method as well as k-means clustering algorithm to calculate and classify the vulnerability degree of agricultural drought. ArcGIS was used to show the spatial and temporal changes of agricultural drought vulnerability in China, then, using the contribution model to analyze the influencing factors and the degrees of agricultural drought vulnerability in China, the results show that:

(1) From 1978 to 2018, the vulnerability of agriculture to drought has been reduced and the numbers of China’s highly vulnerable cities have declined. During the same time, there has been a trend appeared that high vulnerability cities have converted to the middle-level vulnerability cities while middle-level vulnerability cities have converted to mild-level or low-level vulnerability cities. The vulnerability towards agricultural drought disasters in China was generally at the middle and mild level in most regions while the vulnerability in Northwest China and Southwest China were more severe.
(2) China’s agricultural drought vulnerability is mainly affected by sensitivity factors, among which multiple-crop index and the proportion of rural population have a higher contribution compared with other indicators. For resilience index, forest coverage rate and real GDP per capita carry a more important role.

In the data collection process of this paper, partially due to the wide time span selected, there is a lack of data from early years. Therefore, those crucial indicators that can be easily obtained with clean data have been selected for evaluation. Imperfection still exists although these selected indicators can truly reflect the vulnerability characteristics of agricultural drought in China. In the future, we will do some comparative studies on different evaluation methods to further optimize the research results.

## Figures and Tables

**Table 2:** Descriptive statistical results of samples.

Variable	Obs	Mean	Std. Dev.	Min	Max
Agriculture in GDP proportion	279	20.51542	12.75119	0.3193709	59.28663
Multiple-crop index	279	1.371218	0.5034035	0.5117678	2.589842
Rural population proportion	279	60.47917	20.75162	10.39337	91.76649
Annual average temperature	279	13.00551	5.695829	0.5178571	25.18
Annual sunshine duration	279	2136.61	481.6672	703.8	3075.392
Annual precipitation	279	917.2576	495.0083	80.34242	2523
The forest coverage rate	279	23.74077	16.91269	0.3	66.8
Net income per capita of rural residents	279	4124.196	5357.99	100.93	30374.73
Food production per capita	279	377.0782	225.9848	15.84958	1989.61
Real GDP per capita	279	18,178.2	25,786.47	175	140,000
The effective irrigation rate	279	0.5104425	0.229718	0.0719334	1
Agricultural fertilizer per unit area	279	0.0388566	0.0252265	0.002069	0.1870795

**Table 3:** The weight of each indicator.

First-Level Indicator	Weight	Second-Level Indicatorand the Direction of Influence	Weight
A. Sensitivity	0.594	A1. Agriculture in GDP proportion (+)	0.099
		A2. Multiple-crop index (+)	0.145
		A3. Rural population proportion (+)	0.071
		A4. Annual average temperature (+)	0.106
		A5. Annual sunshine duration (+)	0.081
		A6. Annual precipitation (-)	0.091
B. Resilience	0.406	B1. The forest coverage rate (-)	0.102
		B2. Net income per capita of rural residents (-)	0.049
		B3. Food production per capita (-)	0.049
		B4. Real GDP per capita (-)	0.045
		B5. The effective irrigation rate (-)	0.101
		B6. Agricultural fertilizer per unit area (-)	0.061

**Table 4:** Vulnerability of agricultural drought in different provinces.

Province	Years									Level	Sort
	1978	1983	1988	1993	1998	2003	2008	2013	2018		
Shanghai	0.492	0.476	0.491	0.438	0.419	0.378	0.426	0.469	0.303	0.432	1
Beijing	0.505	0.517	0.492	0.513	0.427	0.417	0.399	0.408	0.541	0.469	2
Zhejiang	0.579	0.553	0.407	0.528	0.502	0.443	0.419	0.417	0.375	0.469	3
Guangdong	0.499	0.476	0.501	0.474	0.545	0.500	0.446	0.464	0.345	0.472	4
Fujian	0.549	0.520	0.524	0.521	0.485	0.434	0.467	0.444	0.349	0.477	5
Tianjin	0.514	0.536	0.508	0.522	0.493	0.434	0.460	0.457	0.483	0.490	6
Jilin	0.503	0.491	0.493	0.508	0.515	0.487	0.510	0.492	0.535	0.504	7
Heilongjiang	0.485	0.500	0.493	0.501	0.499	0.479	0.526	0.493	0.562	0.504	8
Liaoning	0.520	0.520	0.534	0.535	0.509	0.494	0.521	0.509	0.582	0.525	9
Jiangsu	0.593	0.612	0.609	0.558	0.526	0.442	0.524	0.530	0.392	0.532	10
Hunan	0.526	0.527	0.554	0.539	0.576	0.508	0.592	0.585	0.398	0.534	11
Jiangxi	0.598	0.571	0.598	0.591	0.547	0.496	0.577	0.538	0.422	0.549	12
Shaanxi	0.590	0.581	0.572	0.570	0.560	0.529	0.586	0.573	0.545	0.567	13
Hubei	0.607	0.587	0.630	0.610	0.585	0.512	0.585	0.574	0.463	0.573	14
Sichuan	0.562	0.581	0.568	0.572	0.650	0.572	0.635	0.616	0.516	0.586	15
Guangxi	0.613	0.636	0.645	0.638	0.587	0.573	0.574	0.581	0.507	0.595	16
Shandong	0.663	0.662	0.667	0.622	0.587	0.528	0.567	0.578	0.495	0.597	17
Inner Mongolia	0.635	0.638	0.634	0.615	0.613	0.568	0.558	0.548	0.579	0.599	18
Shanxi	0.623	0.628	0.615	0.621	0.607	0.551	0.582	0.580	0.598	0.601	19
Hebei	0.639	0.662	0.640	0.632	0.592	0.556	0.589	0.593	0.543	0.605	20
Anhui	0.699	0.646	0.681	0.630	0.612	0.533	0.603	0.585	0.462	0.606	21
Yunnan	0.608	0.614	0.616	0.631	0.614	0.588	0.607	0.642	0.577	0.611	22
Xinjiang	0.634	0.633	0.612	0.587	0.581	0.599	0.634	0.633	0.601	0.613	23
Chongqing	0.674	0.666	0.675	0.681	0.616	0.618	0.583	0.588	0.484	0.620	24
Qinghai	0.583	0.621	0.614	0.618	0.636	0.598	0.619	0.648	0.649	0.621	25
Henan	0.690	0.681	0.710	0.669	0.653	0.543	0.676	0.622	0.483	0.636	26
Hainan	0.657	0.710	0.683	0.649	0.705	0.606	0.584	0.576	0.586	0.640	27
Tibet	0.615	0.674	0.681	0.678	0.647	0.606	0.597	0.610	0.694	0.645	28
Guizhou	0.634	0.659	0.699	0.659	0.734	0.593	0.619	0.641	0.583	0.647	29
Ningxia	0.656	0.686	0.679	0.669	0.648	0.615	0.644	0.622	0.643	0.651	30
Gansu	0.629	0.660	0.645	0.667	0.687	0.643	0.674	0.681	0.735	0.669	31

**Table 5:** Classification of agricultural drought vulnerability.

Grade	Range
Low vulnerability	(0, 0.463)
Mild vulnerability	(0.463, 0.552)
Middle vulnerability	(0.552, 0.628)
High vulnerability	(0.628, 1)

**Table 6:** The top four indicators and contribution of agricultural drought vulnerability in different regions.

Regions	North China		Northeast China		East China		Central and Southern China		Southwest China		Northwest China	
Years												
1978	A3 19.14	A1 17.33	A2 17.69	A3 15.33	A5 16.78	B4 13.51	A5 25.73	A6 19.51	A5 26.02	A3 17.52	A2 30.17	A4 24.05
	A2 15.34	B4 13.98	A4 15.03	A1 12.26	B5 13.39	A6 11.64	B6 12.95	B5 11.22	A2 16.48	A6 12.99	A1 18.47	B6 10.72
1983	A1 16.04	A6 15.46	A2 16.01	A3 14.30	A5 21.32	B5 15.00	A5 32.92	B6 17.32	A5 32.83	A6 12.67	A2 26.31	A6 22.12
	A2 14.50	A3 13.29	A4 13.60	B1 10.88	B4 14.01	A1 10.93	B1 14.13	B5 13.64	A3 12.60	A2 11.74	A4 20.80	A1 12.16
1988	A1 15.47	A3 15.17	A2 16.48	A3 14.72	A5 15.04	B2 13.80	A5 29.65	B6 16.36	A5 33.60	A3 19.91	A2 21.14	A6 19.18
	A2 13.92	B4 12.48	A4 14.00	A1 13.28	B5 13.03	B4 12.94	B1 14.00	A3 12.46	A2 11.73	A4 11.01	A4 17.64	A3 14.32
1993	A1 15.51	A2 15.33	A2 16.44	A4 13.97	A5 20.21	B4 14.89	A5 27.93	B1 15.28	A5 31.28	A3 26.59	A2 21.74	A6 19.60
	A6 13.82	B4 12.51	A3 12.92	A1 11.21	A1 13.02	B2 12.97	B6 15.26	B5 10.7	A6 10.58	A4 10.54	A4 18.43	A1 10.98
1998	A1 14.71	A2 14.60	A2 17.43	A3 15.57	A5 19.33	A1 13.14	A5 29.65	B1 17.82	A5 40.25	A4 14.11	A2 24.87	A6 21.12
	A3 12.84	A6 12.15	A4 14.81	B1 11.85	B2 11.48	B1 10.88	B6 15.27	B5 10.36	A6 11.14	A2 9.93	A4 19.22	A3 18.46
2003	A3 15.25	A2 13.85	A2 16.24	A3 15.17	A5 16.10	B4 12.34	A5 31.60	B1 21.12	A5 36.68	B1 13.65	A2 28.07	A6 22.37
	A1 13.80	A6 12.06	A4 15.03	B1 12.02	A1 12.30	B2 12.34	B6 15.45	A3 10.32	A6 13.37	A2 16.61	A4 20.78	A3 11.94
2008	A3 15.20	A1 13.76	A2 18.76	A4 15.94	A5 16.13	A3 13.64	A5 31.02	B1 21.16	A5 32.99	A2 21.60	A2 27.46	A6 23.87
	A2 11.95	A6 11.87	A3 14.19	A6 11.90	A1 11.56	B2 11.23	B6 16.46	A3 11.95	B1 14.10	A6 11.82	A4 22.45	A1 9.96
2013	A3 15.30	A1 13.85	A2 19.32	A4 16.41	A3 15.52	A5 14.56	A5 31.32	B1 20.26	A5 28.93	A2 19.64	A2 26.18	A6 25.94
	A6 13.85	A2 11.94	A3 12.89	B1 11.16	A1 13.52	B2 11.92	B6 15.76	A3 11.84	B1 15.33	A6 14.52	A4 20.60	A3 7.40
2018	A3 18.97	A1 17.17	A4 19.15	A6 14.24	A2 21.93	A5 19.19	A5 25.82	A2 25.37	A5 21.36	B4 13.26	A6 15.32	A4 13.55
	A6 15.76	A2 12.10	B4 12.87	A3 12.78	A3 18.00	A1 17.68	B4 12.02	A3 10.09	B2 10.30	A6 10.14	B1 13.06	B4 11.94

## Data Availability

Publicly available datasets were analyzed in this study. This data can be found here: China Statistical Yearbook (http://www.stats.gov.cn/english/Statisticaldata/AnnualData/, accessed on 17 July 2020), China Rural Statistical Yearbook (https://data.cnki.net/trade/yearbook/single/n2019120190?z=z009, accessed on 23 July 2020), China Meteorological Administration Data Network (https://data.cma.cn/, accessed on 28 July 2020).
